# Atrazine and Diuron Effects on Survival, Embryo Development, and Behavior in Larvae and Adult Zebrafish

**DOI:** 10.3389/fphar.2022.841826

**Published:** 2022-04-04

**Authors:** Amanda B. Zaluski, Melissa T. Wiprich, Luiza F. de Almeida, Andressa P. de Azevedo, Carla D. Bonan, Monica R. M. Vianna

**Affiliations:** ^1^ Laboratório de Biologia e Desenvolvimento do Sistema Nervoso, Escola de Ciências da Saúde e da Vida, Pontifícia Universidade Católica do Rio Grande do Sul, Porto Alegre, Brazil; ^2^ Laboratório de Neuroquímica e Psicofarmacologia, Escola de Ciências da Saúde e da Vida, Pontifícia Universidade Católica do Rio Grande do Sul, Porto Alegre, Brazil

**Keywords:** survival, teratogenesis, exploratory behavior, cognition, social interaction, atrazine, diuron, zebrafish

## Abstract

Atrazine and Diuron are widely used herbicides. The use of pesticides contaminates the aquatic environment, threatening biodiversity and non-target organisms such as fish. In this study, we investigated the effects of acute exposure for 96 h hours to atrazine and diuron commercial formulations in zebrafish (*Danio rerio*, wild-type AB) embryos and larvae and adult stages. We observed a significant concentration-dependent survival decrease and hatching delays in animals exposed to both herbicides and in the frequency of malformations compared to the control groups. Morphological defects included cardiac edema, tail reduction, and head malformation. At 7 days post-fertilization (dpf), atrazine exposure resulted in a reduction in the head length at 2, 2.5, and 5 mg/L and increased the ocular distance at 1, 2, 2.5, and 5 mg/L atrazine when compared to controls. At the same age, diuron increased the ocular distance in animals exposed to diuron (1.0 and 1.5 mg/L) and no effects were observed on the head length. We also evaluated a behavioral repertoire in larvae at 7 dpf, and there were no significant differences in distance traveled, mean speed, time in movement, and thigmotaxis for atrazine and diuron when animals were individually placed in a new environment. The cognitive ability of the larvae was tested at 7 dpf for avoidance and optomotor responses, and neither atrazine nor diuron had significant impacts when treated groups were compared to their corresponding controls. Adults’ behavior was evaluated 7 and 8 days after the end of the acute herbicide exposure. Exploration of a new environment and associated anxiety-like parameters, social interaction, and aggressiveness were not altered. Our results highlight the need for further studies on the sublethal effects of both herbicides and the consideration of the effects of commercial formulas vs. isolated active ingredients. It also emphasizes the need to take sublethal effects into consideration when establishing the environmental limits of residues.

## 1 Introduction

The demand for greater agricultural productivity over the last few decades was accompanied by the increased use of agrochemicals, such as herbicides, in pest and weed control. The fate of these substances on the environment depends on several factors, such as the concentration and frequency of use, application methods, and environmental biotic and abiotic characteristics (Rodrigues et al., 2017; Tang et al., 2021). The extensive use of these substances leads to air and soil contamination not only at the planting sites but also in local water bodies and groundwater (Fernandes et al., 2020). The persistence of these substances in the environment and the combined use of more than one agrochemical in the same area result in added contamination hazards. Presently, some notoriously toxic substances are still used in several global areas, contributing to biodiversity threats and environmental pollution (Sharma et al., 2019; Maggi et al., 2020; Tang et al., 2021). Country specific regulatory limits and guideline levels for pesticide residues in drinking water and groundwater are mostly based on animal mortality studies that estimate the median lethal concentration (LC50) (Hamilton et al., 2003), but do not include sublethal teratogenic and behavioral effects that may compromise animals’ endurance.

Atrazine and diuron are globally used herbicides associated with significant threat to the ecosystem and human health, despite their ban from some developed countries. In Brazil, diuron and atrazine are used in pre- and post-emergent control of annual grasses and broadleaf weeds in several crops, mainly in cotton, corn, soybean, sugarcane, and pineapple (Lyer, 2003; Solomon et al., 2008; Švorc et al., 2013). Both are water soluble and can leach from fields to surface and groundwater persisting in the environment and possibly affecting the non-target aquatic species (Pereira et al., 2009; Velki et al., 2017). Increased concern is associated with the current lack of information regarding the effect of recurrent and combined exposure to pesticides and their derivatives in more realistic setups, including their environmental and non-target organism impacts (Dellamatrice and Monteiro 2014).

Diuron [3-(3,4-dichlorophenyl)-1,1-dimethylurea] is a phenylurea herbicide extensively used to control weeds in agriculture, urban, and industrial settings (Liu et al., 2013). Stable to hydrolysis at neutral pH (pH 5–9), it is generally persistent in soil with a half-life of more than 200 days (Tang et al., 2021), reaching water bodies through leaching or surface runoff (Giacomazzi and Cochet 2004; Langeron et al., 2014; Liu et al., 2016) and even atmospheric deposition. Moreover, its main degradation product, 3,4-dichloroaniline, exhibits higher toxicity and increased persistence in soils (Giacomazzi and Cochet 2004; Tasca et al., 2018). Atrazine (2-chloro-4-ethylamino-6-isopropylamino-1,3,5-triazine) is the most widely used triazine herbicide in crops globally and acts as a selective systemic herbicide by inhibiting photosynthesis (Plhalova et al., 2012). Its half-life is around 4 weeks and may persist in the environment for up to 2 years (Liu et al., 2016; Tai et al., 2021; Tang et al., 2021). Atrazine is metabolized to desethyl atrazine (DEA), desisopropyl atrazine (DIA), and diaminochlorotriazine (DACT) through cytochrome P450 enzymes in mammals but its mechanism is not fully understood in other species (Liu et al., 2016; Tai et al., 2021) so that its metabolites cause greater toxicity in non-target species. Both substances are also suggested to act as endocrine disruptors (Amaral, 2014; Wirbisky et al., 2015; Akcha et al., 2016; Horzmann et al., 2021). Diuron and atrazine LC50 for fish are 4.50 and 6.70 mg/L, respectively (Tang et al., 2021).

Zebrafish (*Danio rerio*) is an oviparous species with rapid organogenesis, especially suited for toxicological testing through water exposure in all life stages (Sipes et al., 2011; MacRae and Peterson 2015). This social species has a diverse and complex behavioral repertoire (Gerlai et al., 2000; Kalueff et al., 2013) that can contribute for the identification of sublethal effects of agrochemicals and their underlying cellular and molecular mechanisms.

In Brazil, the National Council for the Environment (CONAMA) establishes, through normative resolution CONAMA 357 (2005), limits and allowed environmental concentrations for the agrochemical’s active principles and their environmental hazard classification. However, commercial formulations, known to have increased toxicity due to synergistic effects between components (Liu et al., 2013; Mansano et al., 2016), are not tested to establish such limits, neither are other biological parameters that can hinder species survival due to long-term effects on development, behavior, and reproduction. Also, while isolated atrazine is listed, for diuron these parameters have not been established in the country. For this reason, we decided to test both in parallel.

This study was designed to evaluate the individual toxicity of commercial formulations of atrazine and diuron on survival, hatching, and malformations in zebrafish early life stages. We also evaluated the behavioral parameters of ecological significance, such as exploration of a new environment, cognitive responses, social interaction, and aggression at different life stages, including larvae and adults.

## 2 Materials and Methods

### 2.1 Animals and Ethics

Zebrafish (wild-type, AB strain) embryos and larvae (0–7 days post-fertilization, dpf) and adults (12–18 months) were used. Animals were obtained from our breeding colony and maintained in recirculating systems (Zebtec, Tecniplast, Italy) with equilibrated filtered water to reach the species standard temperature (28 ± 2°C), pH (7.0–7.5), conductivity (300–700 µS), ammonia (<0.02 mg/L), nitrite (<1 mg/L), nitrate (<50 mg/L), and chloride (0 mg/L) levels. Between 5 and 14 dpf, larvae were fed three times a day with crushed commercial flakes (TetraMin Tropical Flake Fish^®^), once including live paramecium. From 14 dpf, animals were also fed thrice a day and received commercial flakes and brine shrimp (*Artemia salina*) (Westerfield, 2000).

For breeding, female and males (1:2) were placed in breeding tanks (Tecniplast, Italy) overnight, and separated by a transparent barrier that was removed after lights went on, the following morning. The embryos, no more than 4 hpf were collected, sanitized, and randomly assigned to each treatment group or control. All procedures followed the guidelines of the Brazilian Council of Animal Experimentation for Use of Fish in Research (CONCEA, 2016), and all protocols were approved by the Animal Care Committee of the Pontifical Catholic University of Rio Grande do Sul (10136/20—CEUA PUCRS). This study is registered at the Sistema Nacional de Gestão do Patrimônio Genético e Conhecimento Tradicional Associado—SISGEN (Protocol No. AD9D212). We followed the ARRIVE guidelines for reporting *in vivo* experiments (Percie du Sert et al., 2020).

### 2.2 Atrazine and Diuron Acute Exposure

Commercial herbicide formulations of atrazine (AclamadoBR^®^, 500 g/L, 50% purity—SC) and diuron (Diuron Nortox^®^, 500 g/L, 50% purity—SC) were diluted in water from recirculating tank systems (Tecniplast, Italy) and the solutions’ pH was adjusted with sodium hydroxide (NaOH) solution to be within the aforementioned range and was verified daily. Embryos and adults were subjected to acute treatment for 96 h, as follows.

The herbicide concentration range was established based on the recommended working solutions and the environmental limits for atrazine. Experiments with embryos and larvae were performed chronologically first and additional concentrations were included for diuron, as it was not regulated in Brazil, and we aimed to explore a greater set of sampling points. Based on the initial findings, subsequent experiments with adults included the same concentrations for both herbicides. Each herbicide had a dedicated control group, to which treated animals were compared, hereafter called as the corresponding control group.

#### 2.2.1 Larval Treatment

**FIGURE 1:**
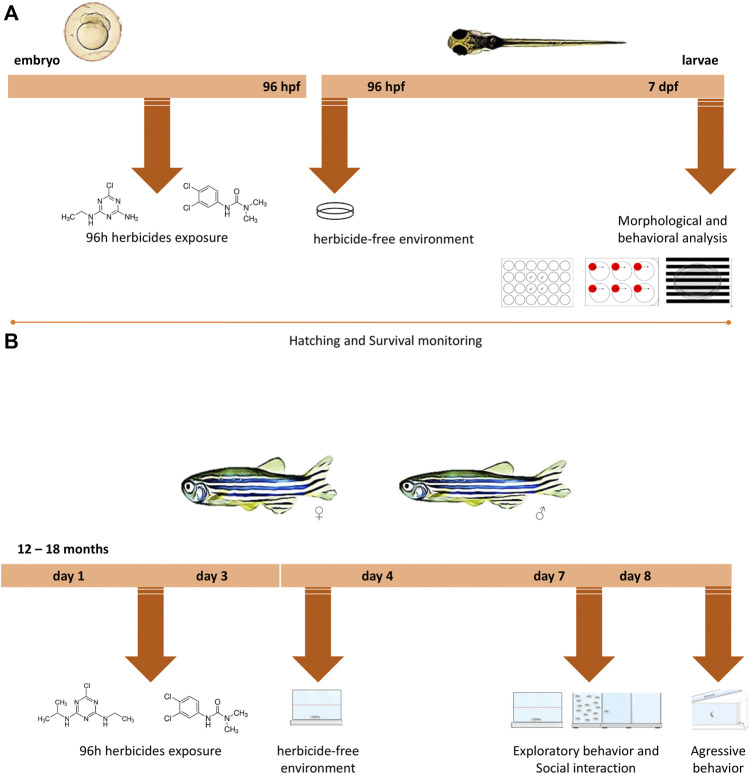
Experimental design timeline for different life stages. **(A)** Zebrafish embryos up to 4 h post-fertilization (hpf) were exposed to water (control groups) or to different concentrations of the two herbicides (atrazine or diuron) for 96 h, after which they were transferred to an environment free of herbicides in which they remained until 7 days post-fertilization (dpf). During 7 dpf, morphological parameters, survival, and hatching rates were daily monitored. At 7 dpf, behavioral analyzes were performed and included the exploration of a new environment, aversive behavior, and optomotor response and individuals were photographed. **(B)** Adult animals aged 12–18 months were exposed to water (control groups) or to different concentrations of atrazine or diuron. Animals were exposed for 96 h, after which they were transferred to an environment free of herbicides where they remained for more than 3 days. On the third day, their performance on a new environment and the social interaction were evaluated. In the following day, an analysis of aggressive behavior was performed.

Embryos up to 4 h post-fertilization (hpf) were placed in Petri dishes (20 embryos per dish, in triplicate) and exposed to concentrations of the isolated herbicides atrazine (0.5, 1.0, 2.0, 2.5, and 5.0 mg/L) or diuron (0.1, 0.5, 1.0, 1.5, 2.0, 2.5, and 5.0 mg/L) for 96 h. After 96 hpf, the embryos were transferred to a pesticide-free environment until 168 hpf, that is, 7 dpf (Figure 1A). The control group went through the same manipulation; however, it was exposed just to the recirculating tank system water (Tecniplast, Italy). The dishes were kept in a biochemical oxygen demand (B.O.D) incubator with a constant temperature of 28°C and light/dark period of 14/10 h.

#### 2.2.2 Adults Treatment

Adult animals, aged between 12 and 18 months of both sexes, were exposed to the concentrations of 0.5, 1.0, 2.0, 2.5, and 5.0 mg/L for both herbicides for 96 h. Animals were maintained in dedicated glass 3L tanks (30 cm long × 15 cm high × 10 cm wide), with aeration at a density of six zebrafish per liter, in triplicate. After the treatment, the animals were transferred to a pesticide-free environment for 3 days until behavioral analysis (Figure 1B). The control group also went through the same manipulation; however, it was exposed just to the recirculating tank system water (Tecniplast, Italy).

### 2.3 Survival and Hatching Rates

Embryos and hatched larvae were daily monitored for 7 dpf for survival defined by the presence of heartbeat and monitored under an inverted stereomicroscope (SMZ 1500 Nikon, Melville, EUA). Daily hatching was determined by the absence of chorion and was expected to occur between 48 and 72 hpf (Westerfield, 2000). The sample size was 60 embryos per group (*n* = 60).

### 2.4 Morphological and Teratogenic Evaluation

Treatment-induced morphological defects were estimated by morphological evaluation of 7 dpf larvae (*n* = 10 in triplicate) under a stereomicroscope (×3 magnification) and included the following parameters: body length (µm), head length (µm), ocular distance (µm), and forebrain and mesencephalon distance (µm), measured after photographic registration using NIS-Elements D software for Windows 3.2 (Nikon Instruments Inc., Melville, United States). Kimmel et al. (1995) staging series were used as a reference from normal development. The body length was defined as the distance from the mouth to the pigmented tip of the tail; the head length was measured from the mouth to the beginning of the pectoral fins; the ocular distance was the distance between the inner edge of the two eyes (Lutte et al., 2015; Wiprich et al., 2020), and the forebrain and mesencephalon width were measured in a coronal body orientation.

### 2.5 Larval Behavioral Analysis

All larvae behavioral experiments were conducted between 11 a.m. and 5 p.m. in a temperature-controlled room (27 ± 2°C) (Altenhofen et al., 2017a; Wiprich et al., 2020). Data quantification and analysis were performed automatedly using EthoVision XT tracking software (version 11.5, Noldus) and by experimenter’s blind-to-individuals’ group assignment.

#### 2.5.1 Exploratory Behavior

At 7 dpf, larvae with no morphological defects were used for general exploratory and locomotion analysis (9 individuals per group, in triplicate) on a protocol adapted from Colwill and Creton (2011; Nabinger et al., 2018; Wiprich et al., 2020). Larvae were individually placed in a 24-well cell culture dish filled with 2 ml of recirculating tank system water (Tecniplast, Italy) and video recorded for automated analysis using EthoVision XT tracking software (version 11.5, Noldus) for 6 min, in a designed protocol that virtually divided each 15 mm diameter well in the inside (7.5 mm diameter) and outside areas (7.5 mm diameter) (Wiprich et al., 2020). Exploratory behavior was analyzed for 5 min after the 1-min acclimatization (Colwill and Creton 2011; Wiprich et al., 2020), and the video-tracking data was used to determine the following parameters: total distance traveled (m) and velocity (m/s, the ratio between distance traveled and movement), and were considered as parameters of exploration of the new environment. The parameter movement was defined as the period during which the zebrafish exceeded the start velocity (defined as 0.06 cm/s) and remained moving until reaching the stop velocity (defined as 0.01 cm/s; Mahmood et al., 2013). The anxiety-like behavior was also measured, and the time spent in each well position (outside area vs. inside area) was considered an index of anxiety. This task exploits the natural tendency of zebrafish to spend most of the time in the outside area when introduced to a novel environment, and then, the animals gradually extend the swimming range to include the inside portion of the test well. A longer time spent in the outside area and the less time spent in the inside of the well indicate increased anxiety (Colwill and Creton 2011).

#### 2.5.2 Avoidance Behavior

The avoidance behavior evaluates animals’ ability to escape an aversive stimulus. After the exploratory behavior, larvae were placed in a 6-well plate (5 larvae per well, in triplicate, per group) over an LCD monitor for the estimation of their avoidance behavior from an aversive visual stimulus (Pelkowski et al., 2011; Nery et al., 2014; Nabinger et al., 2018) for a 5-min session following 2 min of acclimation. A red bouncing ball (1.35 cm diameter) traveled from left to right over a straight 2 cm trajectory under one half of the well (stimulus area), which could be avoided by swimming to the other (non-stimulus) half. The number of larvae in the non-stimulus area was counted every 20 s during the 5-min session and was considered indicative of their cognitive ability of escaping an aversive stimulus (response of escape). Data are reported as the mean percentage of individuals from each group on each trial in the non-stimulus area.

#### 2.5.3 Optomotor Behavior

The optomotor response is a visually driven behavior, in which larvae orientate and move their bodies according to the direction of white and black moving stripes (24.5 cm wide and 1.5 cm high) adapted from Creton (2009) (Nery et al., 2017; Nabinger et al., 2020). Seven dpf larvae (15 larvae per dish, in triplicate, per group) were placed in a Petri dish over an LCD monitor, in which a sequence of animated images of moving stripes were presented for 10 min, following 2 min of acclimation. The stripes moved in alternating directions every 1 min, separated by a blank white screen that lasted 5 s. At the end of each min, when the blank background was presented, animals position inside the well was analyzed. Cognitively and visually apt individuals were expected to follow the stripes movement and therefor to be positioned at the end of the dish into which the stripes were moving. Data are expressed as the mean percentage of individuals from each group on each trial positioned on the far end into which sense the stripes were moving.

### 2.6 Adults Behavioral Analysis

All adult behavioral analyses were performed in a temperature-controlled room (27 ± 2°C) between 8:30 a.m. and 1:00 p.m. to avoid overlapping with their feeding schedule and unspecific effects on performance (Nabinger et al., 2018; Wiprich et al., 2020). Data quantification and analysis were performed automatedly using EthoVision XT tracking software (version 11.5, Noldus) or by experimenter’s blind-to-individuals’ group assignment.

#### 2.6.1 Exploratory Behavior

Three days after the end of the 96 h treatment, each adult animal (six animals per group, in triplicate) was placed individually in experimental tanks (30 cm long × 15 cm high × 10 cm wide) with the recirculating tank system water (Tecniplast, Italy) and the locomotion and exploratory behavior was recorded for 6 min. After 1 min of habituation, the subsequent 5 min was analyzed by using EthoVision XT software (version 11.5, Noldus). The following behavioral parameters were analyzed: distance traveled (m), velocity (m/s, the ratio between distance traveled and movement), time in movement (s), and time spent in upper half of the water column (upper zone) (s). When zebrafish are introduced into a new environment, especially when isolated from their shoal, they tend to spend more time at the bottom of the tank until gradually moving to the upper zone (Levin et al., 2007). Increased time at the half bottom of the water column is indicative of an anxiety-like behavior (Cachat et al., 2010). Time mobile was defined as the period during which animals exceeded the start velocity (0.6 cm/s) and remained moving until reaching the stop velocity (0.59 cm/s), (Tran et al., 2016).

#### 2.6.2 Social Interaction Test

Adults’ social interaction was evaluated 3 days after the end of 96-h acute treatment (6 animals per group, in triplicate), with the same animals used to evaluate exploratory behavior, immediately after, in the same tank to minimize the manipulation interference. Zebrafish are schooling fish that may exhibit a preference for their conspecifics under certain circumstances (Breder and Halpern, 1946; Gerlai et al., 2000). For this, each fish remained individually placed in the experimental tanks (30 cm long × 15 cm high × 10 cm wide). On each smaller side wall of the experimental tank was a glass tank, identically sized (10 cm long × 15 cm high × 10 cm wide): one without fish and one with six adult zebrafish, the latter designated as the “stimulus tank.” To quantify social interaction and innate preference for conspecifics, the experimental tank was virtually divided into three parts: a “stimulus zone” closer to the “stimulus tank” and a “non-stimulus zone” closer to the empty tank, separated by a third central one of equal size. Individual preferences between the stimulus tank zone and the non-stimulus tank zone were automatedly analyzed using EthoVision XT^®^ tracking software (version 11.5, Noldus) (Gusso et al., 2021). Data are presented as mean time (s) spent by individuals from each group in the stimulus zone.

#### 2.6.3 Aggression Test

Four days after the end of 96-h acute treatment, that is, on the day following the exploratory behavior and social interaction tests to ensure experiments were performed within the same time-window for all groups, aggressive behavior was estimated in adult animals (6 animals per group, in triplicate) using the mirror test according to the procedure described by Gerlai et al. (2000) and adapted by Wiprich et al. (2020). Each fish was individually placed in an experimental tank (30 cm long × 15 cm high × 10 cm wide). A mirror (45 cm long) was placed outside the tank at an angle of 22.5° from the 30 cm long wall so that the left vertical edge of the mirror touched the side of the tank, and the right edge was further away. Thus, when the experimental fish swam to the left side of the tank, their mirror image appeared closer to them. After 1-min acclimatization, a 5 min session was recorded for subsequent quantification of aggression behaviors using EthoVision XT tracking software (version 11.5, Noldus). Virtual vertical lines were used to divide the tank into six equally sized sections to allow the investigators to record the number of entries the fish made into each section. Entry to the left-most segment, the sixth part closer to the mirror, indicated a preference for proximity to the “opponent,” whereas entry to the right-most segments implied avoidance. This segment was designated as the stimulus zone. The amount of time the experimental fish spent in each segment was measured as the number of bites against the mirror image, another parameter of aggression. Data are presented as mean time (s) spent by individuals in the stimulus zone, closer to the mirror and the mean number of bites from each group.

### 2.7 Statistical Analysis

Data analyses were performed using Graphpad Prism software version 8.0 (GraphPad Software, Inc.). The data were checked for normality prior to further analysis by the Shapiro–Wilk normality test. Larval survival and hatching rates during the initial 7 dpf were examined with the Kaplan–Meier test. Data from multiple groups of the same herbicide and their respective controls were compared by ANOVA followed by the Tukey’s *post-hoc* test. The data are presented as the mean ± standard error of the mean (S.E.M), except for larval survival and hatching rates that are presented as percentage. For all comparisons, the significance level was set at *p* < 0.05. Statistical differences are graphically indicated as follows: * represents significant differences at *p* ≤ 0.05, ** at *p* ≤ 0.01, *** at *p* ≤ 0.001, and **** at *p* ≤ 0.0001 in relation to the corresponding control group.

## 3 Results

### 3.1 Survival and Hatching Rates

**FIGURE 2:**
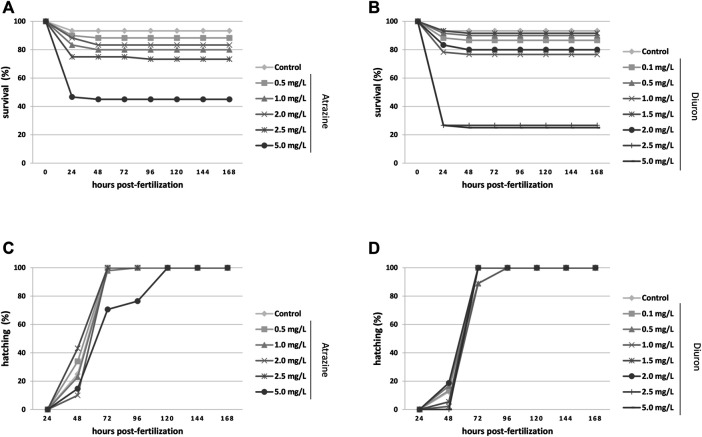
Kaplan–Meier survival and hatching rates for zebrafish embryos and larvae exposed to the herbicides atrazine and diuron during their first 96 hpf and monitored for 7 days (168 hpf). **(A)** Atrazine effects on survival rates. Atrazine significantly reduced survival (*p* < 0.0001) at 5 mg/L when compared to controls; **(B)** Diuron effects on survival rates. Diuron significantly reduced survival (*p* < 0.0001) at 2.5 and 5 mg/L when compared to controls; **(C)** atrazine effects on the hatching rate. Atrazine did not impact hatching on surviving animals (*p* = 0.1231); **(D)** diuron effects on the hatching rate. Diuron significantly delayed hatching (*p* = 0.0359). Rates are expressed as percentage of the total number of animals from each group. Experiments were performed in triplicate, *n* = 60.

The survival and hatching rates were evaluated from 4 hpf until 168 hpf, that is, 7 dpf, and analyzed by the Kaplan–Meier test. Exposure to atrazine commercial formula decreased the survival rate when all groups were compared (*p* < 0.0001, *n* = 60). The control group showed more than 90% survival, as expected for animals from our breeding colony, while only 46% of the animals exposed to 5 mg/L atrazine survived, mostly died at 24 hpf (Figure 2A). Diuron commercial formula also caused a significant decrease in the survival rate (*p* < 0.0001, *n* = 60) when all groups were compared. Dedicated controls also had a 93% of survival rate, whereas animals exposed to 2.5 and 5 mg/L diuron showed 26 and 25% of survival, respectively (Figure 2C).

There was no significant difference in the hatching rate of surviving embryos exposed to atrazine (*p* = 0.1231, *n* = 60). Most groups hatched between 48 and 96 hpf, as expected for this species (Kimmel et al., 1995; Westerfield, 2000), whereas in animals exposed to 5 mg/L atrazine hatching spanned for more than 96 hpf (Figure 2B). The hatching rate was significantly impacted when all groups were compared (*p* = 0.0359, *n* = 60), delayed in groups exposed to the herbicide at 2.5 and 5 mg/L in relation to controls. Whereas most animals started hatching at 48 hpf, and animals exposed to 2.5 and 5 mg/L hatched at 72 hpf (Figure 2D).

### 3.2 Morphology and Teratogenic Evaluation

**FIGURE 3:**
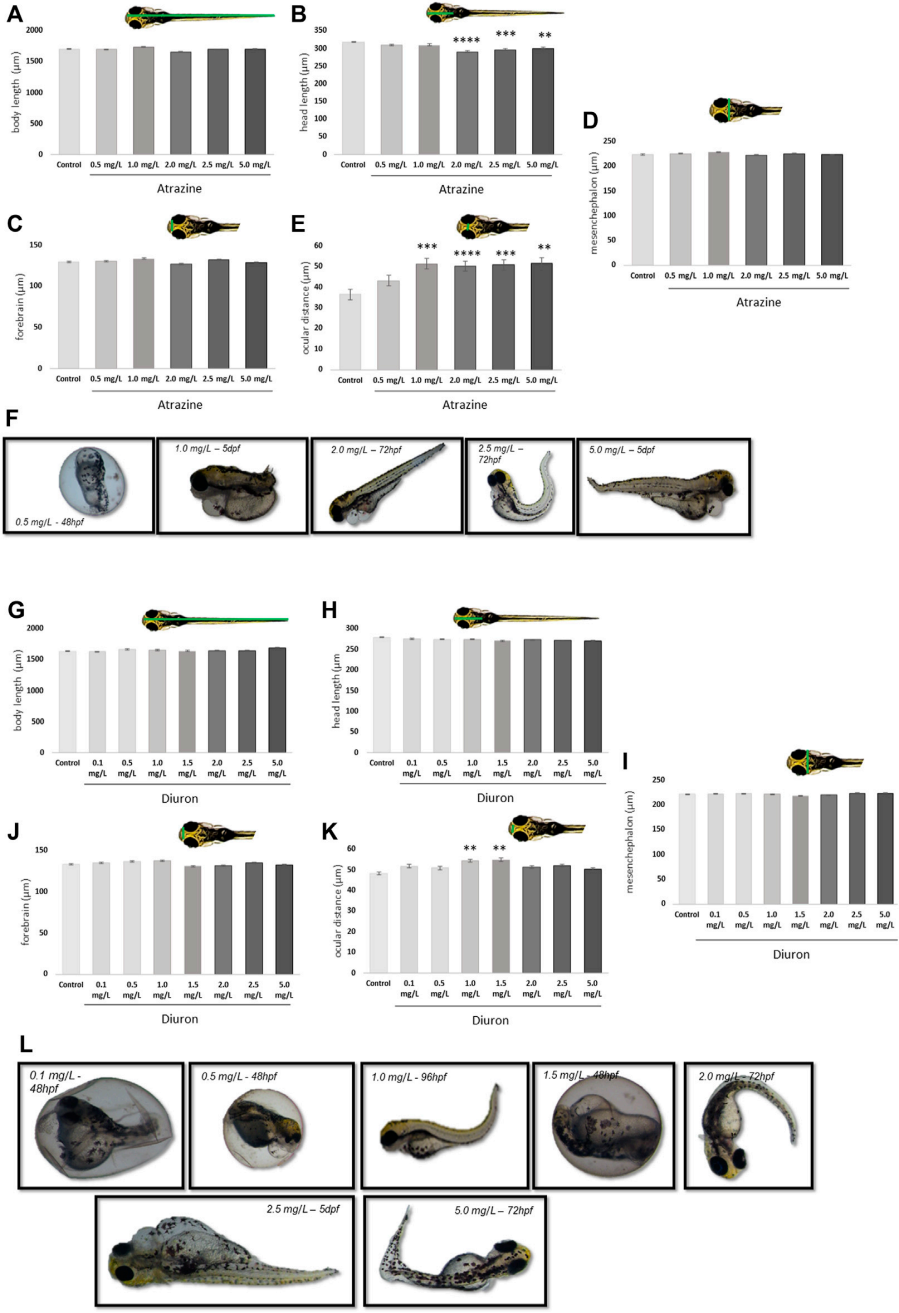
Morphological effects of exposure to the herbicides atrazine and diuron during their first 96 hpf in zebrafish larvae at 7 dpf. **(A)** Atrazine exposure effects on the body length (µm); **(B)** atrazine exposure effects on the head length (µm); **(C)** atrazine exposure effects on the mesencephalon width (µm); **(D)** atrazine exposure effects on the forebrain width (µm); **(E)** atrazine exposure effects on the ocular distance (µm); **(F)** representative images of the most prominent morphology or teratogenic effects observed in individuals exposed to atrazine during the 7 dpf; **(G)** diuron exposure effects on the body length (µm); **(H)** diuron exposure effects on the head length (µm); **(I)** diuron exposure effects on the mesencephalon width (µm); **(J)** diuron exposure effects on the forebrain width (µm); **(K)** diuron exposure effects on the ocular distance (µm); **(L)** representative images of the most prominent morphology or teratogenic effects observed in individuals exposed to diuron during 7 dpf. Data were analyzed by one-way ANOVA followed by a *post-hoc* Tukey’s test. ** represents significant differences at *p* ≤ 0.01, ***p ≤ 0.001, and ****p ≤ 0.0001 in relation to control.

The teratogenic effects of atrazine and diuron herbicides were daily evaluated over 7 dpf in embryos and larvae previously exposed to the herbicides for the initial 96 hpf and photographed at 7 dpf for quantification. For both herbicides, we observed a concentration-dependent increase in the incidence of deformations on body shape, including severe edema of the yolk sac, yolk sac deformation, reduced tail, and absence of head at all concentrations tested, while controls had 1.6% individuals with minor malformation, 0.5, 1.0, and 2.0 mg/L atrazine exposed groups had 5% of altered individuals, 2.5 mg/L had 8% and 5.0 mg/L had 10% (Figure 3F). Similarly, for diuron, controls only had one altered animal (1.6%) while the increasing concentrations of 0.1, 0.5, 1.0, 1.5, 2.0, 2.5, and 5 mg/L also had a progressively increased incidence of 3.3, 5, 6.6, 6.6, 11.6, and 15%, respectively (Figure 3L).

Quantification of discrete morphological parameters at 7 dpf showed that exposure to both herbicides significantly induced malformations on the head structure and brain vesicles, between treatment concentrations and in relation to their dedicated controls.

The body length was different between 1.0 and 2.0 mg/L groups exposed to atrazine (*p* = 0.0005). Atrazine resulted in a reduction in the head length at 2, 2.5, and 5 mg/L at *p* < 0.0001, *p* = 0.0001, and *p* = 0.00027 (F_(5,114)_ = 9.307), respectively, when compared to controls (Figure 3A). The head length also differed between 2.0 mg/L and 0.5 and 1.0 mg/L groups and *p* = 0.0017 and 0.0012, respectively (Figure 3B). A significant increase in the ocular distance in all atrazine groups, except the lower concentration, was observed when compared to controls at *p* < 0.0001 (F_(5,97)_ = 11.85) was observed (Figure 3E). No differences between groups were observed in mesencephalon (*p* = 0.1543; F_(5,114)_ = 1.643) and forebrain (*p* = 0.1027; F_(5,114)_ = 1.883) dimensions (Figures 3C,D).

Animals exposed to diuron also had effects in morphological parameters. The body length differed between 1.0 and 5.0 mg/L groups at *p* = 0.0177 (Figure 3G). The ocular distance was increased in animals exposed to diuron 1.0 and 1.5 mg/L at *p* = 0.0016 and *p* = 0.0006 (F_(7,216)_ = 4.164), respectively (Figure 3K). This parameter was also increased in animals exposed to 1.5 mg/L in relation to 5.0 mg/L, suggesting an inverted U concentration-response curve. Forebrain dimensions were significantly reduced in animals exposed to 1.5 mg/L diuron when compared to 1.0 mg/L (*p* = 0.0432) (Figure 3J). The head length was not significantly impacted in animals exposed to diuron (*p* = 0.7072, F_(7,216)_ = 0.6582) or the mesencephalon dimensions (*p* = 0.5762; F_(7,216)_ = 0.8143) (Figures 3H,I).

### 3.3 Behavior Analysis in Larvae

#### 3.3.1 Exploratory and Locomotor Behavior

**FIGURE 4:**
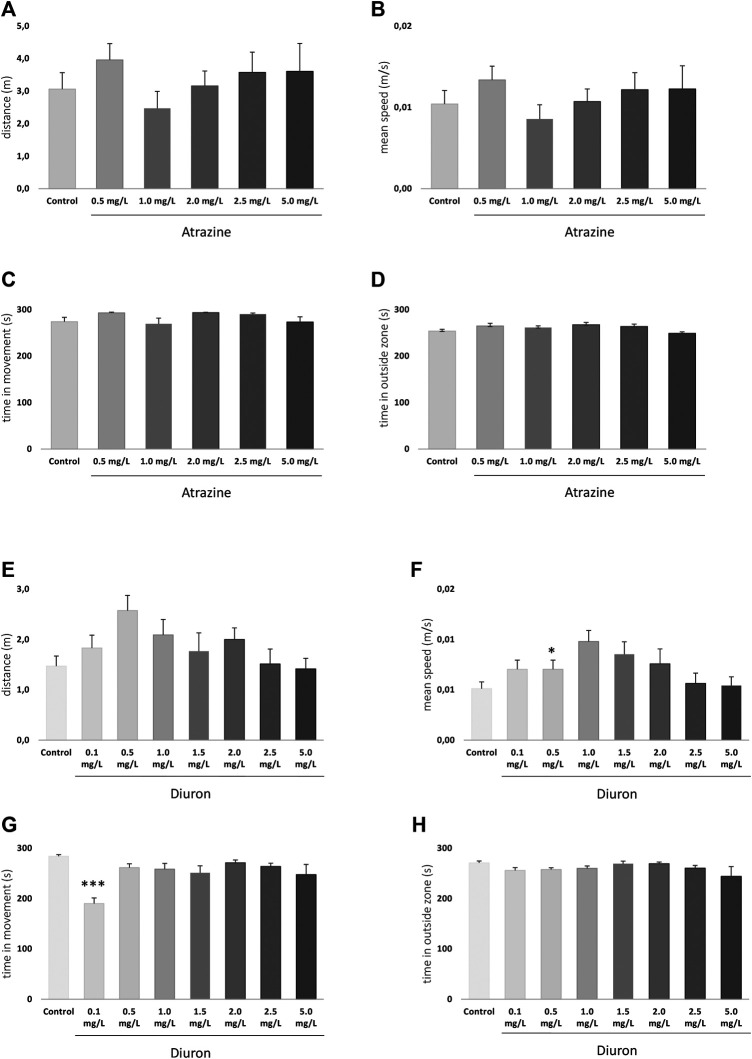
Exploratory behavior of zebrafish larvae at 7 dpf after atrazine or diuron exposure during the initial 96 hpf. Columns depict means ± S.E.M. Sample sizes are *n* = 27 for each group. **(A)** Atrazine exposure effects on total distance traveled (m); **(B)** atrazine exposure effects on mean speed (m/s); **(C)** atrazine exposure effects on time in movement (s); **(D)** atrazine exposure effects on time in the outer area (s) of the well; **(E)** diuron exposure effects on total distance traveled (m); **(F)** diuron exposure effects on mean speed (m/s); **(G)** diuron exposure effects on time in movement (s); **(H)** diuron exposure effects on time in the outside area (s). Data were analyzed by one-way ANOVA followed by a *post-hoc* Tukey’s test. Asterisks represent significant differences at *p ≤ 0.01 and ***p ≤ 0.001 in relation to controls.

The exploratory behavior of larvae in a new environment was analyzed at 7 dpf to determine whether atrazine or diuron exposure could alter larvae locomotion and anxiety. For atrazine, there were no significant differences in distance traveled (m) (*p* = 0.5597; F_(5,155)_ = 0.7881) (Figure 4A), mean speed (m/s) (*p* = 0.5934; F_(5,155)_ = 0.7415) (Figure 4B), time in movement (s) (*p* = 0.8542; F_(5,154)_ = 1.982) (Figure 4C), and the time spent outside the well area (s) (*p* = 0.2765; F_(5,154)_ = 1.277) when all groups were compared (Figure 4D). Despite the lack of statistical significance, when the mean distance traveled in controls (3.1 m) is contrasted with treated groups, an increase is observed in all tested concentrations (4, 3.2, 3.6, and 3.6 m in 0.5, 1.5, 2.0, 2.5, and 5.0 mgL, respectively), except 1.0 mg/L (2.5 m). The same pattern was observed regarding the mean speed.

For diuron, a similar inverted U pattern of mean total distance traveled, and speed is seen (Figures 4E,F) mostly due to the increased values on the lower concentrations in these parameters. Statistically significant differences were seen in the total distance traveled between 0.1 and 0.5 mg/L groups (*p* = 0.0309), and mean speed between 0.5 mg/L and controls (*p* = 0.0309). Time in movement was increased in diuron 0.1 mg/L in relation to controls (*p* = 0.0021) (Figure 4G). No differences were observed between groups regarding the time spent in the outside area (*p* = 0.1388; F_(7,208)_ = 1.594) (Figure 4H).

#### 3.3.2 Avoidance Behavior

**FIGURE 5:**
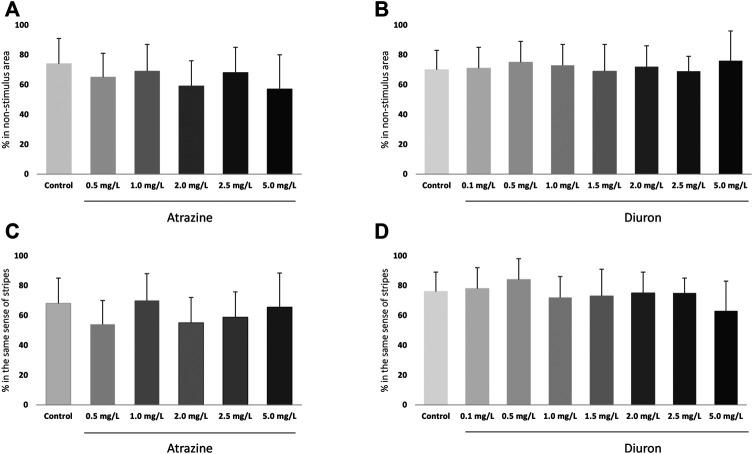
Cognitive effects of atrazine or diuron exposure during the initial 96 hpf at 7 dpf larvae. Columns depict means ± S.E.M. Sample sizes are *n* = 45 for each group. **(A)** Atrazine exposure effects on the avoidance from an aversive visual stimulus; **(B)** Diuron exposure effects on the avoidance from an aversive visual stimulus; **(C)** Atrazine exposure effects on the optomotor response to moving stripes; **(D)** Diuron exposure effects on effects on the optomotor response to moving stripes. Data were analyzed by one-way ANOVA followed by a *post-hoc* Tukey’s test.

Avoidance from a red bouncing ball was evaluated at 7 dpf to test individuals’ cognitive ability to escape an aversive stimulus and the effects of herbicide exposure. There were no changes in avoidance responses after acute exposure to atrazine (*p* = 0.0798; F_(5,48)_ = 2.114) or diuron (*p* = 0.6342; F_(7,61)_ = 0.7459) when exposed groups were compared to their corresponding controls (Figures 5A,B). Nonetheless, when mean responses of herbicide-exposed groups were examined in relation to their controls, that escaped the stimulus in average 74–75%, larvae exposed to all herbicide concentrations showed a lower mean ability to escape the stimulus and move to the non-stimulus zone.

#### 3.3.3 Optomotor Response

The optomotor response to black and white moving stripes in alternating directions was analyzed at 7 dpf. There were no changes on optomotor response after acute exposure to atrazine (*p* = 0.1137; (F_(5,12)_ = 2.269)) and diuron (*p* = 0.4399; (F_(7,15)_ = 1.049)) when exposed groups were compared to their corresponding controls. Animals from all groups showed a positive optomotor response, following the same direction as the stripes, both up and down (Figures 5C,D).

### 3.4 Behavior Analysis in Adults

#### 3.4.1 Exploratory

**FIGURE 6:**
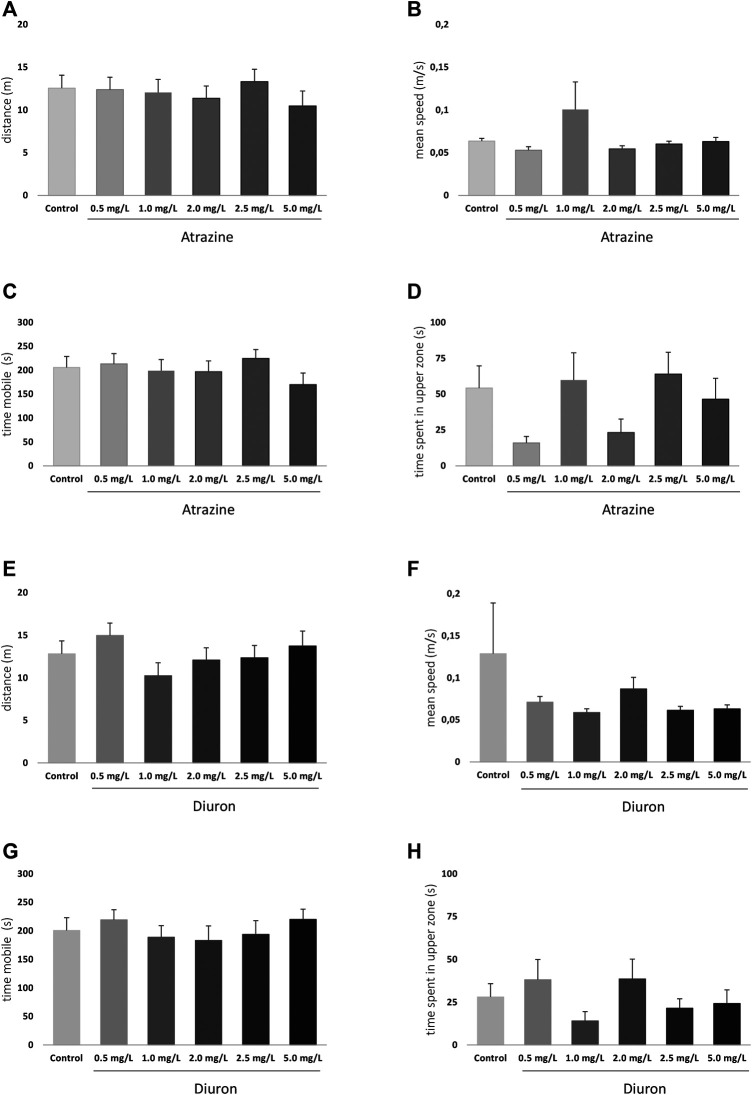
Effects of acute exposure to atrazine or diuron on locomotion and exploratory behavior in adult zebrafish. Columns depict means ± S.E.M. Sample sizes are *n* = 18 for each group **(A)** Atrazine exposure effects on total distance traveled (m); **(B)** atrazine exposure effects on mean speed (m/s); **(C)** atrazine exposure effects on time mobile (s); **(D)** atrazine exposure effects on time spent in the upper tank zone (s); **(E)** diuron exposure effects on total distance traveled (m); **(F)** diuron exposure effects mean speed (m/s); **(G)** diuron exposure effects time mobile (s); **(H)** diuron exposure effects time spent in the upper zone (s). Data were analyzed by one-way ANOVA followed by a *post-hoc* Tukey’s test.

The exploratory and swimming pattern of adult animals in a new environment was analyzed 3 days after the end of the 96 h acute exposure to herbicides. There were no significant differences at any analyzed parameter when herbicide-exposed groups were compared to their dedicated controls. When atrazine groups and their controls were compared, the herbicide exposure did not impact the total traveled distance (*p* = 0.8351, F_(5,118)=_ 0.4185) (Figure 6A), means speed (*p* = 0.4441; F_(5,118)_ = 0.9620) (Figure 6B), time mobile (*p* = 0.6198; F_(5,118)_ = 0.7063) (Figure 6C), and not the time spent in the upper zone of the tank (*p* = 0.0929; F_(5,118)_ = 1.945) (Figure 6D).

For diuron, a similar pattern was observed. Distance traveled (*p* = 0.4484; F_(5,121)_ = 0.9572), (Figure 6E) mean speed (*p* = 0.4011; F_(5,121)_ = 1.034) (Figure 6F), time mobile (*p* = 0.7554; F_(5,121)_ = 0.5271) (Figure 6G), and time in the upper area (*p* = 0.3300; F_(5,121)_ = 1.166) (Figure 6H) were not statistically different between groups. The mean time spent in the upper tank zone varied between groups, but probably due to dispersion, the comparisons were not statistically significant.

#### 3.4.2 Social Interaction

**FIGURE 7:**
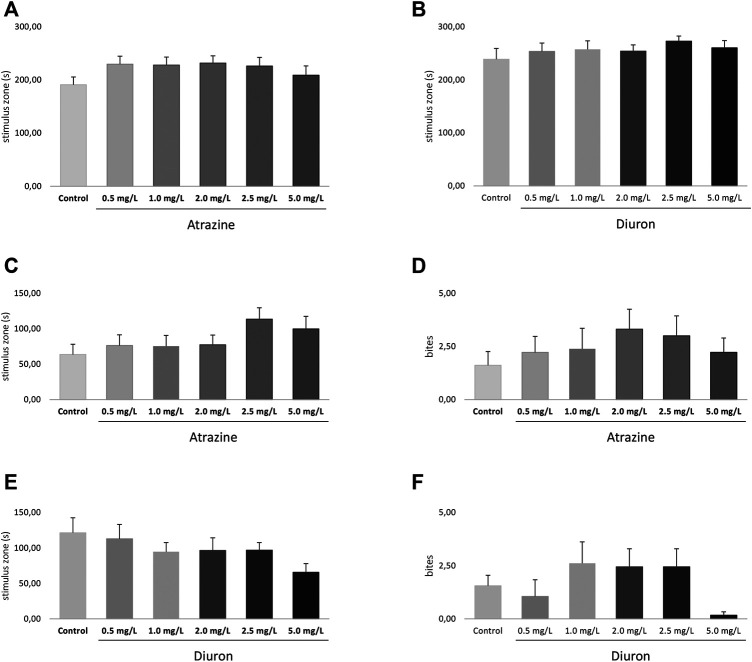
Effects of acute exposure to atrazine or diuron on aggression and social interaction in adult zebrafish. Columns depict means ± S.E.M. Sample sizes are *n* = 18 for each group. **(A)** Atrazine exposure effects on aggressive behavior estimated by the time spent at the stimulus zone closer to the mirror (s); **(B)** atrazine exposure effects on the number of bites; **(C)** diuron exposure effects on aggressive behavior estimated by the time spent at the stimulus zone closer to the mirror (s); **(D)** diuron exposure effects on the number of bites; atrazine exposure effects on time mobile (s); **(E)** atrazine exposure effects on social behavior estimated by the time spent at the stimulus zone closer to the mirror (s); **(F)** atrazine exposure effects on social behavior estimated by the time spent at the stimulus zone closer to the mirror (s). Data were analyzed by one-way ANOVA followed by a *post-hoc* Tukey’s test.

Social interaction was evaluated after the exploratory behavioral test, also 3 days after the end of the 96-h exposure to atrazine and diuron herbicides, in the same apparatus. Atrazine and diuron acute exposure at all concentrations tested did not induce any social interaction deficits measured by the time spent in the stimulus zone when all groups were compared (*p* = 0.5900; F_(5,120)_ = 0.7468 and *p* = 0.7297; F_(5,118)_ = 0.5610) for atrazine and diuron, respectively. Despite the lack of significant effect, a subtle increase is observed in all herbicide-exposed groups in relation to the mean responses of their dedicated controls (Figures 7A,B).

#### 3.4.3 Aggression

Aggressive behavior was analyzed 4 days after the end of 96 h exposure to herbicides and two parameters were used, the time spent in most proximity of the mirror (stimulus zone) and the number of bites directed to their own reflection in the mirror. There were no significant differences in the time spent in the stimulus zone for atrazine (*p* = 0.2250, F_(5,94)=_1.418) (Figure 7C) and diuron (*p* = 0.2268, F_(5,98)=_1.411) (Figure 7E) when all groups are compared. The number of bites were also not statistically different between atrazine (*p* = 0.7520; F_(5,100)_ = 0.5314) (Figure 7D) and diuron (*p* = 0.1742; F_(5,102)_ = 1.574) (Figure 7F) exposed groups and their controls.

Despite the lack of statistical effect, animals exposed to all concentrations tested of atrazine spent in average more time in the segment nearest to the mirror when compared to the control group and showed an increased average number of biting episodes. In turn, for diuron-exposed groups, the average time spent in the stimulus zone was lower than that of controls, while the number of bites fluctuated.

## 4 Discussion

Non-target species contamination by herbicides in aquatic environments occurs as a result of leaching, direct spraying, or during heavy rainfall of agricultural fields (Roy et al., 2016; Bridi et al., 2017). In the last years, Brazil was the country that had the highest consumption of pesticides and that most approved new commercial formulations in a few months (Ferrari et al., 2014; Frota et al., 2021). Most of these formulations have not been tested or regulated by CONAMA, so they do not have residue limit levels despite current use. For this reason, we analyzed, in a wide range of decreasing concentrations in relation to the known fish LC50 for the active principles of each, two herbicides: atrazine, a regulated pesticide, and diuron, currently unregulated in Brazil. In Brazil, it has been reported that diuron and atrazine herbicides are the second and the fourth most used herbicides, respectively, mostly found in surface waters, mainly near sugarcane crops (Bortoluzzi et al., 2006; Britto et al., 2011) at concentrations much higher than those allowed for the triazine group, and that should not exceed 2.0 μg/L (CONAMA 357, 2005).

Atrazine and diuron usage concentrations differ in the literature, but most studies with animal models and zebrafish use the isolated substance with >92% purity, which is not representative of the potential impacts due to the well reported synergistic toxicity between principle and vehicle substances (Corvi et al., 2012; Wang et al., 2015; Akcha et al., 2016; Liu et al., 2016; Velki et al., 2017, 2019; Horzmann et al., 2021; Tai et al., 2021). This is, to our knowledge, one of the very few studies that assess sublethal effects using commercial formulation as starter solution for treatment preparation, which is expected to be more realistic in estimating potential contamination effects on off-target organisms and environment.

Atrazine has a long history of toxicological tests and debatable toxicity, and data for model organisms vary according to species, developmental stage, exposure duration, concentrations, and formulation used. For diuron, however, there is a shortage of studies in non-target species such as fish (Velki et al., 2017), especially in adults, but also in embryos, and larvae zebrafish (Velki et al., 2017; Velki et al., 2019; 1314 Kao et al., 2018). Other species such as Nile tilapia *Oreochromis niloticus*) (Felício et al., 2017; Boscolo et al., 2018) and Javanese medaka also have a very limited number of studies (*Oryzias javanicus*) (Ibrahim et al., 2020).

Importantly, we observed a significant concentration-dependent survival decrease and hatching delays in animals exposed to both herbicides (Figure 2). Our survival curves are in accordance with the LC50 range for both substances, and differences may result from species and formulations used, in addition to manipulation. Our findings agree with Velki et al. (2017), that found 96 h-LC50 values of 6.31 ± 0.19 mg/L for zebrafish embryos exposed to diuron non-commercial formulation on the FET assay. Walker et al. (2018), also using isolated atrazine, found atrazine-induced mortality in an equivalent time window, but at lower concentrations.

The hatching delays observed are in accordance with previous reports using the isolated atrazine pure principle at lower concentrations (Liu et al., 2016; Walker et al., 2018). The interrelation between survival and hatching rates may hinder some deleterious effects that are not evident in the later parameter, as the lack of hatching delay in 5.0 mg/L atrazine-exposed animals only includes the surviving half population and may be considered when interpreting data.

Morphological defects and malformations were also more prevalent in herbicide-exposed groups than controls and included cardiac edema, tail reduction, and absence of head. The malformation types were found to agree with those expected for the species embryotoxicity tests (Beekhuijzen et al., 2015). The low incidence of malformations observed is typically seen in control animals from our AB wild-type breeding colony (Velki et al., 2017; Horzmann et al., 2021; Tai et al., 2021) and may vary depending on the vehicle solution controls exposed to, but are in accordance to other studies testing atrazine toxicity in similar and lower concentrations (Liu et al., 2016). Our findings are also in agreement with Blahova et al. (2020) that tested a wide range of environmentally relevant atrazine concentrations, using the isolated principle and found a significant increase in the occurrence of pericardial edema in the 10 mg/L group in relation to controls. They also paralleled those from Velki et al. (2017) which also observed body defects induced by pure diuron at 1.0, 2.0, and 3.8 mg/L.

In addition to a concentration-dependent effect on the frequency of malformations, specific morphological differences were observed between herbicide-exposed groups and their controls. The ocular distance was the most affected parameter, followed by the head length. Atrazine had more robust morphological effects than diuron, inducing reduced head length and increased ocular distance at several concentrations, while diuron increased the ocular distance in specific groups. Increased ocular distance may be associated with several factors, including changes in overall head morphology and reduced ocular dimensions. Craniofacial abnormalities resulting from the developmental defects on chondrogenesis and osteogenesis may underly these effects, as atrazine exposure was previously shown to result in craniofacial defects in zebrafish embryos (Walker et al., 2018), but it has not been associated with diuron and deserve further investigation. The observed decrease in the head length and increase in the ocular distance may also be associated with the brain developmental effects of the herbicide exposure. Horzmann et al. (2021) demonstrated that early atrazine exposure results in neurotoxic changes in adult males, behavioral changes and anxiety, and cellular density on raphe brain cell population months after exposure.

Even discrete morphological effects may have lasting impacts on individuals’ behavioral performance and physiology, endangering animals through their life cycle. To try to separate the morphological effects from exploratory and cognitive impacts of herbicide exposure, we only included individuals free from morphological defects on the behavioral analysis of 7 dpf larvae. This, of course, resulted in less pronounced behavioral effects than if abnormal animals were included, but may be strategic when looking for discrete effects (Velki et al., 2017). Despite the resulting lack of effect on exploratory parameters, traveled distances and mean speeds for each set of herbicide-exposed groups show a similar profile, as can be observed in Figure 4. Additionally, no effect was observed regarding thigmotaxis, suggesting anxiety was not impacted under these conditions and that has not been tested in other studies.

We also investigated exploratory parameters of 7 dpf larvae in a new environment. Swimming and responding to threats are critical abilities for larval fish, as is responding to visual cues. We did not observe any significant impact on exploratory and cognitive responses under tested conditions. The lack of effect is not in agreement with Liu et al (2016) that found reduced locomotion in zebrafish larvae after exposure to 100 and 300 ug/L atrazine. Velki et al (2017) also found an increase in the total distance moved by zebrafish larvae 118 h after exposure to diuron at concentrations of 1 and 2 mg/L, but not 3 mg/L, in a sudden light–dark transition test. Despite statistically significant, their effects were very subtle and may be related to the specific task, in which sudden transitions in lighting foster behavioral changes. These differences may be related to exposure conditions and may be attributed to research suggesting that the effects of exposure to atrazine are reversible once the exposure ends (Solomon et al., 2008).

Despite a reduced cognitive repertoire in comparison to adults, zebrafish larvae show specific visual-driven cognitive responses. We used previously established and validated protocols (Pelkowski et al., 2011; Nery et al., 2014, 2017; Nabinger et al., 2018; Nabinger et al., 2020) to measure aversive and optomotor responses in 7 dpf larvae exposed to atrazine and diuron. No significant differences were observed when treated animals were compared to their corresponding controls. Studies conducted with other pesticides showed a decrease in response to a visual stimulus, for glyphosate and Roundup^®^ (Bridi et al., 2017) and the tebuconazole insecticide (Altenhofen et al., 2017b). Differences may be attributed to the different substances, underlying mechanisms and concentrations tested.

Adult zebrafish has a well-characterized behavioral repertoire (Kalueff et al., 2013) and consistent protocols to evaluate them under experimental conditions (Nery et al., 2014, 2017; Altenhofen et al., 2017a; Bridi et al., 2017; Nabinger et al., 2018; Nabinger et al., 2020; Wiprich et al., 2020; Gusso et al., 2021). This is, however, the first study assessing the acute toxic effects of diuron on the adult zebrafish behavior. We chose to evaluate ecologically relevant sets of behaviors: exploration of a new environment and associated anxiety-like parameters, social interaction, and aggressiveness. We did not find any significant effect on exploratory parameters and anxiety when they were individually placed in a new environment, while studies investigating the effects of diuron on behavior of zebrafish are scarce, diuron influences on the behavior of other fish have been assessed, that is, goldfish observed a higher burst swimming activity after exposure to diuron (Saglio and Trijasse 1998).

Adult zebrafish are social and interdependent of their school conspecifics. Social behavior, including aggression, social interaction, dominance, and inter-dependence tends to be impacted by separation from their conspecifics (Pagnussat et al., 2013). Under the conditions tested, we did not observe significant impacts of herbicide exposure on the parameters of social interaction. Our data diverges from the decreased social interaction found by Schmidel et al. (2014), showing increased inter-fish distance and an overall shoal area after 1.0 mg/L atrazine exposure. Differences between their and our findings regarding the social interaction may be attributed to the treatment regimen, which was subchronic and lasted 14 days, in contrast to ours that only lasted 4 days.

The lack of behavioral effects does not the exempt these substances from inducing other deleterious impacts. Sposito et al. (2018) demonstrated that atrazine and diuron on ng/L concentration range could alter zebrafish embryos gene expression after 3 days of exposure beginning at 48 hpf. Importantly, our findings agree to the expected increased susceptibility of early life stages, parallel to increased tolerance of adults. However, it is important to consider the possible long-term effects of the early life exposure in adults, which was not tested here, but were reported by Horzmann et al. (2021). In their study, they demonstrated that embryonic atrazine exposure during the initial 72 hpf decreases locomotor activity in males and alters gene expression.

## 5 Conclusion

This study investigated toxicological effects of atrazine and diuron exposure in sublethal concentrations—below the estimated LC50 for fish—and found impacts of survival, hatching, and morphology of zebrafish embryos, and larvae. These effects may be further explored to characterize its underlying mechanisms and potential impacts in other outcomes that can hinder animals’ health span and lead to significant population decline.

Despite the lack of significant effects on behavioral outcomes in larvae and adult individuals, future studies may deepen the behavioral characterization of the herbicides’ effects of fish, as behavioral changes may represent advantageous endpoints to estimate sublethal toxicity and fishes are vulnerable to the environmental contamination by agrochemicals.

## Data Availability

The raw data supporting the conclusion of this article will be made available by the authors, without undue reservation.
